# Body weight–normalized resting metabolic rate and its associations with malnutrition and sarcopenia in hospitalized older adults: a cross-sectional study

**DOI:** 10.3389/fnut.2026.1914763

**Published:** 2026-10-12

**Authors:** Shan Jiang, Jie Zhao, Peng Chen, Lehui Chen, Weihong Chen, Zi Lin, Lin Kang, Hailong Li, Xia Zhao, Qiumei Wang

**Affiliations:** 1 Department of Geriatrics, Peking Union Medical College Hospital, Chinese Academy of Medical Sciences & Peking Union Medical College, Dongcheng, Beijing, China; 2 Department of Geriatrics, Handan Central Hospital, Hanshan, Handan, Hebei, China; 3 Department of Geriatric Endocrinology, The First Affiliated Hospital of Zhengzhou University, Erqi, Zhengzhou, Henan, China; 4 Department of Geriatrics, Hangzhou Wuyunshan Hospital, Xihu, Hangzhou, Zhejiang, China; 5 Department of Geriatrics, Beijing Sixth Hospital, Dongcheng, Beijing, China; 6 Department of Geriatrics, The Affiliated People's Hospital of Fujian University of Traditional Chinese Medicine, Fuzhou, Fujian, China; 7 Department of Clinical Nutrition, Peking Union Medical College Hospital, Chinese Academy of Medical Sciences & Peking Union Medical College, Dongcheng, Beijing, China

**Keywords:** GLIM, indirect calorimetry, malnutrition, older adults, resting metabolic rate, sarcopenia

## Abstract

**Background:**

Malnutrition and sarcopenia are major contributors to frailty, functional decline, and mortality in older adults. Although the Global Leadership Initiative on Malnutrition (GLIM) criteria provide a standardized framework for diagnosing malnutrition, they do not incorporate physiological measures of metabolic function. We therefore investigated the associations of body weight–normalized resting metabolic rate (RMR/weight) with GLIM-defined malnutrition and sarcopenia in hospitalized older adults.

**Methods:**

This cross-sectional study analyzed baseline data from a prospective cohort of hospitalized older adults who underwent comprehensive geriatric assessment (CGA) at Peking Union Medical College Hospital (PUMCH) between February 2024 and August 2025. Malnutrition was diagnosed according to the GLIM criteria, and sarcopenia was defined according to the Asian Working Group for Sarcopenia (AWGS) 2019 criteria. RMR was measured by indirect calorimetry and normalized to body weight (kcal/kg/day). Associations between RMR/weight and study outcomes were evaluated using multivariable logistic regression. Receiver operating characteristic (ROC) analysis was performed to assess the discriminatory ability of RMR/weight for malnutrition.

**Results:**

A total of 141 participants (mean age, 74.8 ± 6.6 years; 54.6% women) were included, of whom 74 (52.5%) had GLIM-defined malnutrition. Both RMR and RMR/weight were significantly lower in participants with malnutrition than in those without malnutrition. After adjustment for age, sex, Charlson Comorbidity Index (CCI) score, and Katz Activities of Daily Living (ADL) score, each 1-kcal/kg/day increase in RMR/weight was associated with lower odds of GLIM-defined malnutrition [odds ratio (OR), 0.698; 95% confidence interval (CI), 0.597–0.816; *p* < 0.001] and sarcopenia (OR, 0.756; 95% CI, 0.658–0.870; *p* < 0.001). RMR/weight showed moderate discriminatory ability for malnutrition (area under the curve, 0.759; 95% CI, 0.677–0.838). An exploratory threshold of 20.5 kcal/kg/day yielded a sensitivity of 83.8% and specificity of 40.3%. Low RMR/weight (< 20.5 kcal/kg/day) was associated with higher odds of GLIM-defined malnutrition and sarcopenia, as well as with all three GLIM phenotypic criteria and several sarcopenia components.

**Conclusions:**

Lower RMR/weight was independently associated with GLIM-defined malnutrition and sarcopenia in hospitalized older adults. RMR/weight may provide complementary metabolic information regarding nutrition- and muscle-related impairment, although its clinical utility requires further validation.

## 1 Introduction

Malnutrition is a common and clinically significant condition among older adults, particularly those who are hospitalized or have multiple chronic diseases (1). It is associated with a wide range of adverse health outcomes, including functional decline, prolonged hospital stay, increased risk of complications, and higher mortality (2–4). To improve diagnostic consistency, the Global Leadership Initiative on Malnutrition (GLIM) proposed a standardized framework for diagnosing malnutrition that incorporates both phenotypic criteria, including unintentional weight loss, low body mass index (BMI), and reduced muscle mass, and etiologic criteria, including reduced food intake or assimilation and inflammation or disease burden (5). Despite these advances, the assessment of malnutrition in older adults continues to rely primarily on anthropometric measurements and clinical indicators (6), while the metabolic alterations associated with malnutrition remain incompletely characterized.

Energy metabolism is fundamental to maintaining physiological homeostasis in older adults. Resting metabolic rate (RMR), which represents the energy required to sustain essential physiological functions at rest, accounts for the largest proportion of total daily energy expenditure. RMR is strongly influenced by body composition, particularly fat-free mass (FFM) and skeletal muscle mass (7, 8). Age-related changes in body composition, characterized by progressive loss of skeletal muscle mass and increased adiposity, may therefore contribute to alterations in energy metabolism (9). Lower resting energy expenditure has been associated with muscle loss, frailty, and impaired physical function in older adults (10, 11). However, the relationship between metabolic activity and malnutrition, particularly malnutrition defined according to the GLIM criteria, remains insufficiently understood.

Malnutrition and sarcopenia frequently coexist in older adults (12) and share several pathophysiological features, including loss of muscle mass, chronic inflammation, and impaired physical function, as well as adverse clinical outcomes (13). Because skeletal muscle is a major determinant of RMR, declines in muscle mass and function may substantially influence energy metabolism (9). Accordingly, alterations in RMR may reflect metabolic changes associated with nutritional status and muscle health (14). Normalizing RMR to body weight may provide a measure of resting energy expenditure relative to body size, potentially facilitating comparisons among individuals with different body weights. Nevertheless, evidence remains limited regarding whether body weight–normalized RMR (RMR/weight) is associated with GLIM-defined malnutrition and sarcopenia in hospitalized older adults (15).

Therefore, this study aimed to investigate the associations of RMR/weight with GLIM-defined malnutrition and sarcopenia in hospitalized older adults undergoing comprehensive geriatric assessment (CGA). We also evaluated the discriminatory ability of RMR/weight for malnutrition and explored its associations with individual components of malnutrition and sarcopenia.

## 2 Materials and methods

### 2.1 Study design and patient selection

This study was a cross-sectional analysis of prospectively collected data from hospitalized older adults admitted to the Department of Geriatrics at Peking Union Medical College Hospital (PUMCH) between February 2024 and August 2025. Only baseline measurements obtained during hospitalization were included in the analysis; no longitudinal follow-up data were evaluated. The Department of Geriatrics functions as a geriatric evaluation and management unit that provides multidisciplinary CGA and care for older adults with multiple chronic conditions and functional impairments. Eligible participants met the following inclusion criteria: (1) age ≥60 years; (2) hospital stay ≥7 days; and (3) ability to undergo body composition assessment and indirect calorimetry. The exclusion criteria were acute infection, fever, thyroid dysfunction, active malignancy under treatment, uncontrolled heart failure or renal failure, pacemaker implantation, and terminal illness with a life expectancy of ≤ 6 months.

The study was conducted in accordance with the principles of the Declaration of Helsinki and was approved by the Ethics Committee of PUMCH (approval number: I-24PJ0185; approval date: January 22, 2024). Written informed consent was obtained from all participants before enrollment.

### 2.2 Study procedure and data collection

All assessments were performed by physicians who had completed standardized training in CGA. Data were collected across the domains described below.

#### 2.2.1 Clinical and demographic characteristics

Demographic and anthropometric characteristics, including age, sex, height, body weight, and BMI, were recorded. Medical history included both chronic and acute medical conditions. Comorbidity burden was assessed using the Charlson Comorbidity Index (CCI), which assigns weighted scores to comorbid conditions identified according to International Classification of Diseases (ICD) codes (16).

#### 2.2.2 Functional status assessment

Basic activities of daily living (ADL) were assessed using the Katz Index of Independence in Activities of Daily Living (17). Instrumental activities of daily living (IADL) were evaluated using the Lawton Instrumental Activities of Daily Living Scale (18).

#### 2.2.3 Assessment of malnutrition

Malnutrition was diagnosed using the two-step approach recommended by the GLIM (5). During the screening phase, nutritional risk was assessed using the Mini Nutritional Assessment-Short Form (MNA-SF) (19). Participants with an MNA-SF score ≤ 11 were considered at nutritional risk and proceeded to the diagnostic phase. A diagnosis of malnutrition required the presence of at least one phenotypic criterion (non-volitional weight loss, low BMI, or reduced muscle mass) and at least one etiologic criterion (reduced food intake or assimilation, or inflammation/disease burden), according to the GLIM framework (Table 1).

**Table 1:** GLIM criteria and thresholds used for malnutrition diagnosis.

Diagnostic domain	Criterion	Threshold
Phenotypic criteria	Unintentional weight loss	>5% within the past 6 months or >10% beyond 6 months
	Low body mass index (BMI)	< 18.5 kg/m^2^ for age < 70 years; < 20.0 kg/m^2^ for age ≥70 years
	Reduced muscle mass^*^	Skeletal muscle index < 7.0 kg/m^2^ in men and < 5.7 kg/m^2^ in women
Etiologic criteria	Reduced food intake or assimilation	< 50% of energy requirements for >1 week or any reduction in food intake for >2 weeks; gastrointestinal symptoms or chronic gastrointestinal conditions adversely affecting food intake, digestion, absorption, or assimilation
	Inflammation or disease burden	Acute disease or injury, chronic disease–related inflammation, or hsCRP >5 mg/L

Malnutrition was diagnosed in the presence of at least one phenotypic criterion and at least one etiologic criterion.

BMI, body mass index; GLIM, global leadership initiative on malnutrition; hsCRP, high-sensitivity C-reactive protein.

^*^Reduced muscle mass was defined using the skeletal muscle index cutoffs recommended by the Asian Working Group for Sarcopenia 2019 criteria (20).

#### 2.2.4 Assessment of sarcopenia

Muscle strength was assessed using an electronic handgrip dynamometer (Camry EH101, Guangdong, China). Each hand was tested twice, with a minimum rest interval of 30 s between attempts. The highest value obtained from the four measurements was used for analysis. Physical performance was evaluated using the five-times-sit-to-stand test, 4-m gait speed test, and Short Physical Performance Battery (SPPB). The SPPB comprises balance, gait speed, and chair-stand assessments, each scored from 0 to 4, yielding a total score ranging from 0 to 12, with higher scores indicating better physical performance. Body composition was measured using a multifrequency bioelectrical impedance analyzer (InBody 770, InBody Co., Ltd., Seoul, South Korea). Parameters included fat-free mass (FFM) and skeletal muscle index (SMI). Sarcopenia was diagnosed according to the Asian Working Group for Sarcopenia (AWGS) 2019 criteria (20). Low muscle strength was defined as handgrip strength < 28 kg in men and < 18 kg in women. Poor physical performance was defined as gait speed < 1.0 m/s, five-times-sit-to-stand time ≥12 s, or SPPB score ≤ 9. Low muscle mass was defined as SMI < 7.0 kg/m^2^ in men and < 5.7 kg/m^2^ in women. Sarcopenia was confirmed when low muscle mass coexisted with low muscle strength or poor physical performance.

#### 2.2.5 Measurement of RMR

RMR was measured using a ventilated-canopy indirect calorimetry system (Quark PFT Ergo, COSMED, Rome, Italy). Participants fasted for at least 8 h and rested in a supine position for at least 20 min before testing. Measurements were performed in a thermoneutral environment at 22–25 °C. “Steady state” was defined as a period of at least 5 min during which oxygen consumption (VO_2_) and carbon dioxide production (VCO_2_) varied by < 10%. Participants remained awake, motionless, and supine beneath a transparent acrylic canopy (40-L volume) with a loose seal around the chest throughout the assessment. Gas exchange data were recorded at 1-min intervals for at least 40 min or until 20 consecutive minutes of steady-state measurements had been obtained. RMR (kcal/day) was calculated using the Weir equation, and the respiratory quotient was calculated as the ratio of VCO_2_ to VO_2_. The mean value obtained during the steady-state period was used as the measured RMR.

#### 2.2.6 Derived metabolic variables

RMR/weight was calculated by dividing measured RMR (kcal/day) by body weight (kg) and was expressed as kilocalories per kilogram of body weight per day (kcal/kg/day). RMR/weight was selected as the primary metabolic indicator because it expresses resting energy expenditure relative to body weight and facilitates comparisons among individuals with different body weights. RMR/weight was analyzed as a continuous variable in the primary analyses. For exploratory categorical analyses, participants were classified into low and high RMR/weight groups using a data-derived threshold identified by receiver operating characteristic (ROC) curve analysis.

### 2.3 Statistical analyses

All statistical analyses were performed using SPSS version 26.0 (IBM Corp., Armonk, NY, USA). The sample size was estimated *a priori* for ROC analysis, assuming an area under the ROC curve (AUC) of 0.70 under the alternative hypothesis vs. 0.50 under the null hypothesis, a two-sided α of 0.05, 80% power, and an approximately 1:1 ratio of participants with and without malnutrition. The minimum required sample size was 62; allowing for 10% missing or invalid data, the target sample size was ≥69 participants. The distribution of continuous variables was assessed using the Shapiro–Wilk test. Continuous variables are presented as mean ± standard deviation (SD) or median [interquartile range (IQR)], as appropriate, and were compared between groups using the independent-samples *t*-test or Mann–Whitney *U*-test. Categorical variables are presented as counts and percentages and were compared using the χ^2^ test or Fisher's exact test, as appropriate. Binary logistic regression was used to examine the associations of RMR/weight with GLIM-defined malnutrition and sarcopenia. Primary models were adjusted for age, sex, CCI score, and ADL score, which were selected *a priori* based on their clinical relevance to resting energy expenditure, nutritional status, and sarcopenia. Because ADL may not act solely as a confounder, sensitivity analyses were performed using models that excluded ADL from the adjustment set. In a separate sensitivity analysis, residual RMR was derived from a linear regression of measured RMR on FFM and standardized as a *Z*-score to account for variation in RMR associated with FFM. Results are reported as odds ratios (ORs) with 95% confidence intervals (CIs). ROC analysis was used to evaluate the discriminatory ability of RMR/weight for GLIM-defined malnutrition and to derive an exploratory threshold. The ROC-derived threshold of 20.5 kcal/kg/day was subsequently fixed, and its internal performance was assessed using 1,000 stratified bootstrap resamples, with percentile-based 95% CIs estimated for the AUC, sensitivity, and specificity. Additional adjusted logistic regression analyses examined the associations of low RMR/weight, defined using this exploratory threshold, with malnutrition, sarcopenia, and their individual diagnostic components. All tests were two-sided, and *p* < 0.05 was considered statistically significant.

## 3 Results

### 3.1 Baseline characteristics

During the study period, 222 hospitalized older adults were assessed for eligibility, of whom 81 were excluded. A total of 141 participants were included in the final analysis, with a mean age of 74.8 ± 6.6 years; 77 (54.6%) were women. According to the GLIM criteria, 74 participants (52.5%) were classified as malnourished and 67 (47.5%) as non-malnourished. Compared with participants without malnutrition, those with malnutrition had significantly lower BMI, ADL and IADL scores, handgrip strength, FFM, and SMI (all *p* < 0.05). Both absolute RMR and RMR/weight were significantly lower in participants with malnutrition (both *p* < 0.001). The prevalence of sarcopenia was also significantly higher among participants with malnutrition (70.3% vs. 28.4%, *p* < 0.001). No significant between-group differences were observed in age, sex, CCI score, hsCRP level, five-times-sit-to-stand performance, gait speed, or SPPB score (all *p* > 0.05). Detailed baseline characteristics are presented in Table 2.

**Table 2:** Baseline demographic, functional, body composition, and metabolic characteristics according to GLIM-defined malnutrition status.

Variable	Malnutrition (*n* = 74)	No malnutrition (*n* = 67)	*p*-value^*^
Demographic characteristics			
Age (years), mean (SD)	75.34 (6.70)	74.22 (6.41)	0.316
Women, *n* (%)	37 (50.0)	40 (59.7)	0.248
BMI (kg/m^2^), mean (SD)	20.67 (4.06)	25.38 (3.41)	< 0.001
CCI score, median (IQR)	4.00 (3.00–5.00)	4.00 (3.00–4.00)	0.173
hsCRP (mg/L), median (IQR)	1.11 (0.50–5.51)	1.11 (0.50–4.40)	0.716
Functional and nutritional status			
Katz ADL score, mean (SD)	5.26 (0.98)	5.58 (0.80)	0.034
Lawton IADL score, mean (SD)	6.34 (2.21)	7.07 (1.63)	0.028
MNA-SF score, mean (SD)	7.95 (2.24)	12.60 (1.49)	< 0.001
Handgrip strength (kg), mean (SD)	21.74 (6.72)	24.40 (7.85)	0.032
Men	25.73 (5.53)	31.77 (6.38)	< 0.001
Women	17.20 (4.83)	19.42 (3.73)	0.026
Five-times-sit-to-stand test (s), mean (SD)	14.97 (6.14)	14.36 (6.71)	0.627
Gait speed (m/s), mean (SD)	0.91 (0.25)	0.95 (0.28)	0.413
SPPB score, mean (SD)	8.57 (2.97)	8.87 (3.18)	0.566
Body composition and metabolic parameters			
FFM (kg), mean (SD)	36.61 (9.55)	40.70 (8.34)	0.008
SMI (kg/m^2^), mean (SD)	5.96 (1.24)	6.44 (1.22)	0.027
RMR (kcal/day), mean (SD)	972.86 (240.04)	1,132.37 (241.75)	< 0.001
RMR/weight (kcal/kg/day), mean (SD)	17.50 (3.23)	20.22 (2.44)	< 0.001
Sarcopenia, *n* (%)	52 (70.3)	19 (28.4)	< 0.001

ADL, activities of daily living; BMI, body mass index; CCI, Charlson Comorbidity Index; FFM, fat-free mass; GLIM, global leadership initiative on malnutrition; hsCRP, high-sensitivity C-reactive protein; IADL, instrumental activities of daily living; IQR, interquartile range; MNA-SF, mini nutritional assessment-short form; RMR, resting metabolic rate; SD, standard deviation; SMI, skeletal muscle index; SPPB, short physical performance battery.

^*^Comparisons were performed between participants with and without GLIM-defined malnutrition.

### 3.2 Associations of RMR/weight with malnutrition and sarcopenia

In multivariable logistic regression analyses adjusted for age, sex, CCI score, and ADL score, higher RMR/weight was associated with lower odds of both GLIM-defined malnutrition and sarcopenia. Each 1-kcal/kg/day increase in RMR/weight was associated with 30.2% lower odds of malnutrition (OR, 0.698; 95% CI, 0.597–0.816; *p* < 0.001) and 24.4% lower odds of sarcopenia (OR, 0.756; 95% CI, 0.658–0.870; *p* < 0.001). Sensitivity analyses excluding ADL from the adjustment set yielded similar results for both outcomes (Supplementary Table 1). In a separate sensitivity analysis accounting for FFM-related variation in RMR, higher standardized residual RMR remained associated with lower odds of GLIM-defined malnutrition (adjusted OR, 0.614; 95% CI, 0.420–0.897; *p* = 0.012) and sarcopenia (adjusted OR, 0.545; 95% CI, 0.368–0.807; *p* = 0.002). These sensitivity analyses yielded findings consistent with those of the primary analyses based on RMR/weight.

### 3.3 Discriminatory performance of RMR/weight for GLIM-defined malnutrition

ROC curve analysis demonstrated moderate discriminatory ability of RMR/weight for GLIM-defined malnutrition, with an AUC of 0.759 (95% CI, 0.677–0.838; *p* < 0.001; Figure 1). An exploratory threshold of 20.5 kcal/kg/day was selected based on the observed balance between sensitivity and specificity. At this threshold, sensitivity and specificity were 83.8% and 40.3%, respectively. The corresponding positive predictive value (PPV) and negative predictive value (NPV) were 60.8% and 69.2%, respectively, and the positive and negative likelihood ratios (LR+ and LR–) were 1.40 and 0.40, respectively. The contingency table underlying these estimates is provided in Supplementary Table 2, and diagnostic performance metrics for additional candidate thresholds are presented in Supplementary Table 3. Bootstrap assessment using 1,000 stratified resamples yielded estimates consistent with those observed in the original sample. The mean bootstrap AUC was 0.760 (95% bootstrap CI, 0.672–0.835). At the fixed exploratory threshold of 20.5 kcal/kg/day, the mean bootstrap sensitivity was 83.9% (95% bootstrap CI, 75.7%−91.9%), and the mean bootstrap specificity was 40.1% (95% bootstrap CI, 28.4%−52.2%).

**Figure 1:**
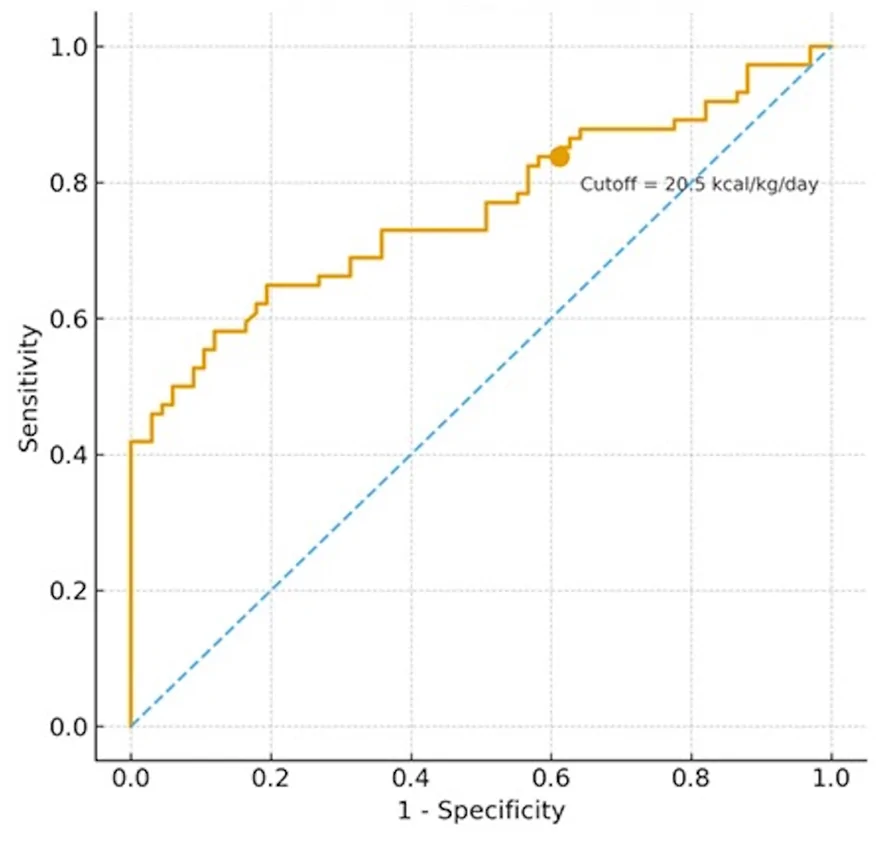
ROC curve of RMR/weight for GLIM-defined malnutrition. The ROC curve illustrates the discriminatory ability of RMR/weight for identifying GLIM-defined malnutrition. The AUC was 0.759 (95% CI, 0.677–0.838; *p* < 0.001). The highlighted point indicates the exploratory threshold of 20.5 kcal/kg/day, which yielded a sensitivity of 83.8% and specificity of 40.3%. The diagonal line represents discrimination expected by chance. AUC, area under the curve; CI, confidence interval; GLIM, global leadership initiative on malnutrition; RMR, resting metabolic rate; ROC, receiver operating characteristic.

### 3.4 Association between low RMR/weight and GLIM-defined malnutrition

For exploratory analyses, participants were categorized into low (< 20.5 kcal/kg/day; *n* = 102) and high (≥20.5 kcal/kg/day; *n* = 39) RMR/weight groups using the threshold derived from the ROC analysis. GLIM-defined malnutrition was more prevalent in the low RMR/weight group than in the high RMR/weight group (60.8% vs. 30.8%, *p* = 0.003). After adjustment for age, sex, CCI score, and ADL score, low RMR/weight was associated with higher odds of GLIM-defined malnutrition (OR, 3.681; 95% CI, 1.625–8.340; *p* = 0.002). Low RMR/weight was also associated with higher odds of weight loss (OR, 4.430; 95% CI, 1.950–10.062; *p* < 0.001), low BMI (OR, 2.831; 95% CI, 1.128–7.102; *p* = 0.027), and reduced muscle mass (OR, 2.724; 95% CI, 1.239–5.991; *p* = 0.013). In contrast, low RMR/weight was not significantly associated with either GLIM etiologic criterion—reduced food intake or assimilation or inflammation or disease burden (both *p* > 0.05; Table 3).

**Table 3:** Multivariable logistic regression analyses of associations between low RMR/weight and malnutrition, sarcopenia, and their components.

Outcome	Adjusted OR (95% CI)	*p*-value
GLIM-defined malnutrition	3.681 (1.625–8.340)	0.002
GLIM phenotypic criteria		
Weight loss	4.430 (1.950–10.062)	< 0.001
Low BMI	2.831 (1.128–7.102)	0.027
Reduced muscle mass	2.724 (1.239–5.991)	0.013
GLIM etiologic criteria		
Reduced food intake or assimilation	1.882 (0.851–4.164)	0.119
Inflammation or disease burden	0.662 (0.295–1.482)	0.316
Sarcopenia	5.602 (2.323–13.506)	< 0.001
Sarcopenia components		
Low handgrip strength	4.445 (1.931–10.234)	< 0.001
Five-times-sit-to-stand test ≥12 s	3.133 (1.325–7.406)	0.009
Gait speed < 1.0 m/s	0.865 (0.354–2.114)	0.751
SPPB score ≤ 9	2.930 (1.198–7.169)	0.019

Low RMR/weight was defined using the exploratory threshold of < 20.5 kcal/kg/day, with RMR/weight ≥20.5 kcal/kg/day as the reference category. All models were adjusted for age, sex, CCI score, and ADL score.

ADL, activities of daily living; BMI, body mass index; CCI, Charlson Comorbidity Index; CI, confidence interval; GLIM, global leadership initiative on malnutrition; OR, odds ratio; RMR, resting metabolic rate; SPPB, short physical performance battery.

### 3.5 Associations between low RMR/weight and sarcopenia components

After adjustment for age, sex, CCI score, and ADL score, low RMR/weight was associated with higher odds of sarcopenia (OR, 5.602; 95% CI, 2.323–13.506; *p* < 0.001). Low RMR/weight was also associated with higher odds of low handgrip strength (OR, 4.445; 95% CI, 1.931–10.234; *p* < 0.001), five-times-sit-to-stand time ≥12 s (OR, 3.133; 95% CI, 1.325–7.406; *p* = 0.009), and SPPB score ≤ 9 (OR, 2.930; 95% CI, 1.198–7.169; *p* = 0.019). In contrast, no significant association was observed with gait speed < 1.0 m/s (OR, 0.865; 95% CI, 0.354–2.114; *p* = 0.751; Table 3 and Figure 2).

**Figure 2:**
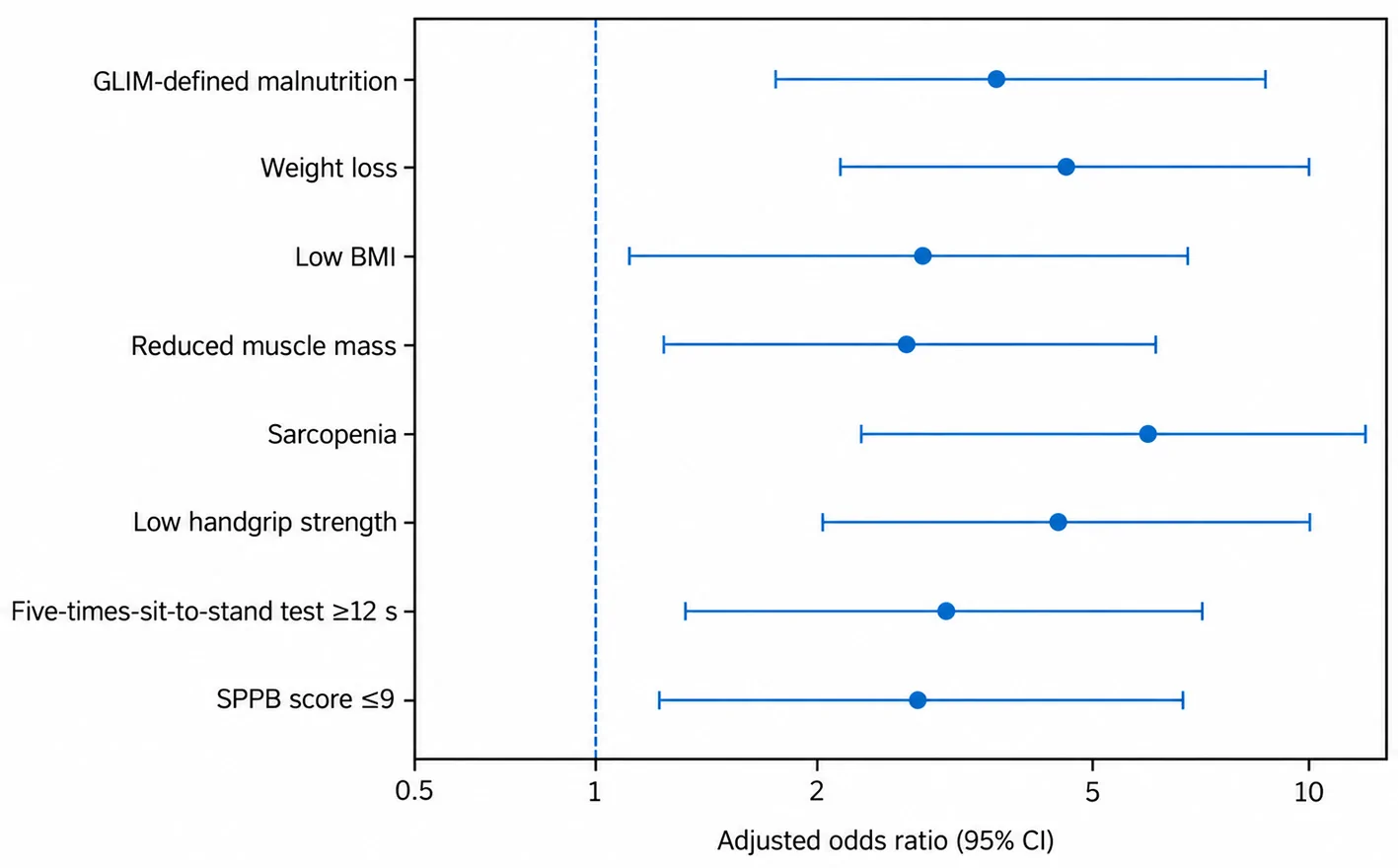
Forest plot of associations between low RMR/weight and selected malnutrition- and sarcopenia-related outcomes. The forest plot shows adjusted ORs and 95% *CIs* for the associations of low RMR/weight, defined using the exploratory threshold of < 20.5 kcal/kg/day, with GLIM-defined malnutrition; the GLIM phenotypic criteria of weight loss, low BMI, and reduced muscle mass; sarcopenia; and selected sarcopenia components. All models were adjusted for age, sex, CCI score, and Katz ADL score. The vertical dashed line indicates an OR of 1.0. ADL, activities of daily living; BMI, body mass index; CCI, Charlson Comorbidity Index; CI, confidence interval; GLIM, global leadership initiative on malnutrition; OR, odds ratio; RMR, resting metabolic rate; SPPB, short physical performance battery.

## 4 Discussion

In this study, lower RMR/weight was independently associated with both GLIM-defined malnutrition and sarcopenia in hospitalized older adults after adjustment for age, sex, comorbidity burden, and functional status. Low RMR/weight was also associated with all three GLIM phenotypic criteria and several sarcopenia-related functional impairments. RMR/weight showed moderate discriminatory ability for malnutrition, although the exploratory threshold of 20.5 kcal/kg/day had limited specificity. Collectively, these findings suggest that lower RMR/weight may represent a metabolic feature accompanying nutritional and muscle-related impairment in hospitalized older adults.

The association between RMR/weight and malnutrition should be interpreted in the context of the close relationship between body composition and resting energy expenditure (21). Malnutrition is frequently accompanied by losses of FFM and skeletal muscle, which are major determinants of RMR (22), while inadequate energy intake and functional decline may coexist with alterations in resting energy expenditure (23, 24). Importantly, the associations with malnutrition and sarcopenia remained significant when FFM-related variation in RMR was accounted for using a residual-based approach. Nevertheless, because RMR/weight incorporates body weight in its denominator, whereas the GLIM phenotypic criteria include weight loss, low BMI, and reduced muscle mass, some degree of shared body-size and body-composition information is inherent in these measures. Consistent with this consideration, low RMR/weight was associated with all three GLIM phenotypic criteria but not with the etiologic criteria of reduced food intake or assimilation and inflammation or disease burden. This pattern suggests that RMR/weight may partly reflect shared body-composition and metabolic characteristics accompanying the phenotypic expression of malnutrition rather than necessarily representing a distinct metabolic abnormality. The residual RMR analysis provides complementary evidence that the observed associations were not entirely attributable to FFM; however, it does not eliminate the potential influence of other body-composition characteristics or establish that RMR/weight reflects a metabolic process independent of nutritional phenotype.

Age-related alterations in cellular energetics may provide additional biological context for these findings. Mitochondrial dysfunction, including impaired oxidative capacity and mitochondrial quality control, may alter cellular energy turnover, while anabolic resistance may impair the skeletal muscle response to nutritional and hormonal stimuli. These processes are interconnected through nutrient- and energy-sensing pathways. In particular, mechanistic target of rapamycin complex 1 (mTORC1) integrates amino acid, insulin/growth-factor, and cellular energy signals to regulate protein synthesis, autophagy, and metabolic homeostasis (25–27), whereas AMP-activated protein kinase (AMPK) is activated during energy stress and promotes energy-conserving catabolic responses (28, 29). Age-related dysregulation of the AMPK–mTORC1 axis, together with impaired sirtuin 1 (SIRT1)/peroxisome proliferator-activated receptor gamma coactivator 1-alpha (PGC-1α) signaling and mitochondrial function, may therefore provide a plausible biological framework for the interrelationships among altered energy metabolism, nutritional impairment, and muscle loss (30, 31). However, these pathways were not directly assessed in our cohort and should be regarded as providing hypothesis-generating biological context rather than causal explanations for the observed associations.

Lower RMR/weight was also independently associated with sarcopenia and with low handgrip strength, prolonged chair-stand performance, and lower SPPB scores, but not with slow gait speed. Previous studies have similarly linked basal metabolic rate and energy balance with sarcopenia, muscle strength, and physical activity in older adults (32–34). Because skeletal muscle is both a major determinant of resting energy expenditure and an important metabolically active tissue, these associations may partly reflect shared relationships among muscle mass, muscle function, and systemic energy metabolism (35). The observed associations of lower RMR/weight with malnutrition and sarcopenia, together with their potential shared biological and metabolic context, are summarized schematically in Figure 3.

**Figure 3:**
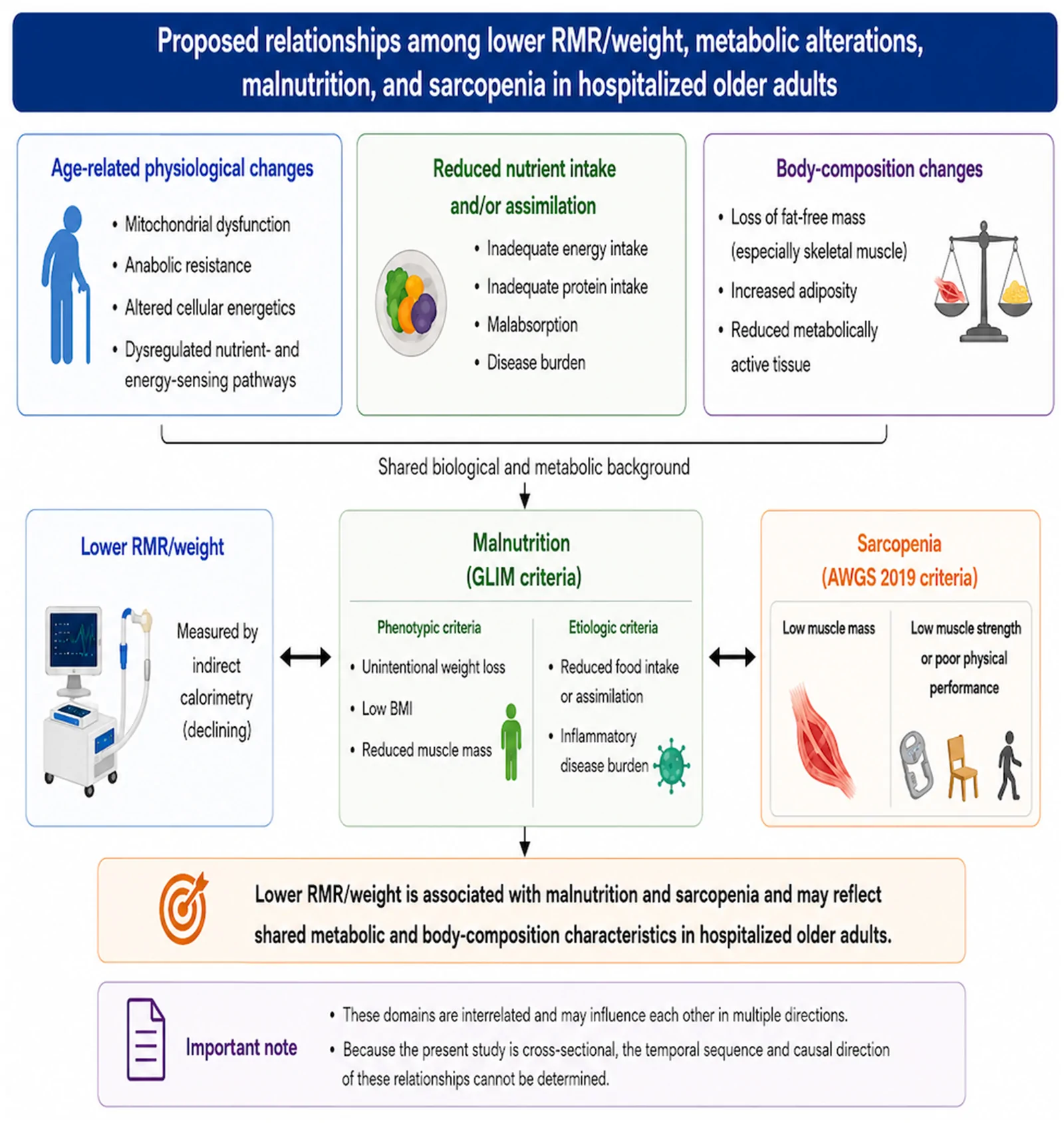
Proposed relationships among lower RMR/weight, metabolic alterations, malnutrition, and sarcopenia in hospitalized older adults. The schematic illustrates the proposed interrelationships among lower RMR/weight, GLIM-defined malnutrition, and AWGS 2019-defined sarcopenia, together with potential shared biological and metabolic factors, including age-related physiological changes, reduced nutrient intake or assimilation, and changes in body composition. Lower RMR/weight was associated with malnutrition and sarcopenia in the present study and may reflect shared metabolic and body-composition characteristics. Given the cross-sectional design, the temporal sequence and causal direction of these relationships cannot be determined. AWGS, Asian Working Group for Sarcopenia; BMI, body mass index; GLIM, global leadership initiative on malnutrition; RMR, resting metabolic rate.

From a clinical perspective, RMR/weight may provide complementary metabolic information alongside established nutritional and functional assessments. Normalization to body weight is readily interpretable because body weight is routinely available and energy requirements are commonly expressed in kcal/kg/day, while indirect calorimetry provides an objective measure of resting energy expenditure that is not directly captured by conventional anthropometric or clinical assessments (36, 37). Nevertheless, the ROC-derived threshold of 20.5 kcal/kg/day should be interpreted cautiously. Although it showed relatively high sensitivity, its low specificity implies a substantial false-positive rate and limits its utility as a stand-alone screening or diagnostic threshold. Because this threshold was derived and evaluated within the same single-center cohort, both the proposed threshold and analyses based on the low/high RMR/weight classification should be regarded as exploratory. Although bootstrap assessment yielded estimates consistent with those observed in the original sample, external validation in independent cohorts is required before this threshold can be considered for clinical application.

Several limitations should be acknowledged. First, the cross-sectional design precludes determination of temporal or causal relationships among RMR/weight, malnutrition, and sarcopenia. Second, the exploratory RMR/weight threshold was derived and evaluated within the same cohort and had limited specificity, requiring external validation before clinical application. Third, residual confounding and the influence of shared body-size and body-composition characteristics on RMR/weight and the GLIM phenotypic criteria cannot be fully excluded, despite consistent findings in the FFM-adjusted and ADL sensitivity analyses. Finally, the modest sample size and single-center setting limit the generalizability of the findings, warranting validation in larger, independent, multicenter cohorts.

## 5 Conclusion

This study showed that RMR normalized by body weight was independently associated with GLIM-defined malnutrition and sarcopenia in hospitalized older adults undergoing CGA. Lower RMR/weight was also associated with adverse nutritional phenotypes and functional impairments, suggesting that it may represent a metabolic feature accompanying malnutrition and sarcopenia in this population. RMR/weight may therefore provide complementary metabolic information alongside established nutritional and functional assessments. Future prospective and longitudinal studies are needed to clarify the temporal relationships among RMR/weight, nutritional status, and functional decline and to determine the clinical utility of RMR-based measures for nutritional assessment and risk stratification.

## Data Availability

The raw data supporting the conclusions of this article will be made available by the authors, without undue reservation.
