# Supplementary material for: Body weight–normalized resting metabolic rate and its associations with malnutrition and sarcopenia in hospitalized older adults: a cross-sectional study
Source: Front Nutr. 2026 Oct 12;13:1914763. doi: 10.3389/fnut.2026.1914763 (PMC13696476; doi:10.3389/fnut.2026.1914763)
Supplement: Supplementary file 1 [file Table_1.docx]

**Supplementary Material**

**Supplementary Table 1. Sequential multivariable logistic regression analyses of the associations of RMR/weight with malnutrition and sarcopenia**

| **Outcome** | **Model** | **OR** | **95% CI** | ***p* value** |
| --- | --- | --- | --- | --- |
| GLIM-defined malnutrition | Model 1 | 0.694 | 0.594–0.811 | <0.001 |
|  | Model 2 | 0.698 | 0.597–0.816 | <0.001 |
| Sarcopenia | Model 1 | 0.751 | 0.654–0.862 | <0.001 |
|  | Model 2 | 0.756 | 0.658–0.870 | <0.001 |

Note: ORs represent the change in the odds of each outcome per 1-kcal/kg/day increase in RMR/weight.
Model 1 was adjusted for age, sex, and CCI score.
Model 2 was adjusted for age, sex, CCI score, and ADL score.
*Abbreviations*: ADL, activities of daily living; CCI, Charlson Comorbidity Index; CI, confidence interval; GLIM, Global Leadership Initiative on Malnutrition; OR, odds ratio; RMR, resting metabolic rate.

**Supplementary Table 2. Contingency table for the exploratory RMR/weight threshold of 20.5 kcal/kg/day**

| **RMR/weight** | **Malnutrition** | **No malnutrition** |
| --- | --- | --- |
| <20.5 kcal/kg/day | 62 | 40 |
| ≥20.5 kcal/kg/day | 12 | 27 |

**Note**: At the exploratory threshold of 20.5 kcal/kg/day, sensitivity was 83.8%, specificity was 40.3%, PPV was 60.8%, NPV was 69.2%, LR+ was 1.40, and LR− was 0.40.

*Abbreviations***:** LR+, positive likelihood ratio; LR−, negative likelihood ratio; NPV, negative predictive value; PPV, positive predictive value; RMR, resting metabolic rate.

**Supplementary Table 3. Performance indices for candidate RMR/weight thresholds for GLIM-defined malnutrition**

| **Threshold (kcal/kg/day)** | **Sensitivity (%)** | **Specificity (%)** | **PPV (%)** | **NPV (%)** | **LR+** | **LR−** |
| --- | --- | --- | --- | --- | --- | --- |
| 17.5 | 52.7 | 89.6 | 84.8 | 63.2 | 5.04 | 0.53 |
| 18.0 | 62.2 | 80.6 | 78.0 | 65.9 | 3.20 | 0.47 |
| 18.5 | 64.9 | 73.1 | 72.7 | 65.3 | 2.41 | 0.48 |
| 19.0 | 66.2 | 71.6 | 72.1 | 65.8 | 2.33 | 0.47 |
| 19.5 | 73.0 | 64.2 | 69.2 | 68.3 | 2.04 | 0.42 |
| 20.0 | 77.0 | 46.3 | 61.3 | 64.6 | 1.43 | 0.50 |
| **20.5** | **83.8** | **40.3** | **60.8** | **69.2** | **1.40** | **0.40** |
| 21.0 | 87.8 | 26.9 | 57.0 | 66.7 | 1.20 | 0.45 |
| 21.5 | 89.2 | 22.4 | 55.9 | 65.2 | 1.15 | 0.48 |
| 22.0 | 91.9 | 17.9 | 55.3 | 66.7 | 1.12 | 0.45 |

**Note:** The exploratory threshold selected for the primary categorical analyses is shown in bold.

*Abbreviations*: GLIM, Global Leadership Initiative on Malnutrition; LR+, positive likelihood ratio; LR−, negative likelihood ratio; NPV, negative predictive value; PPV, positive predictive value; RMR, resting metabolic rate.
